# Suppressing the Aging Phenotype of Mesenchymal Stromal Cells: Are We Ready for Clinical Translation?

**DOI:** 10.3390/biomedicines12122811

**Published:** 2024-12-11

**Authors:** Ilaria Roato, Matteo Visca, Federico Mussano

**Affiliations:** Department of Surgical Sciences, CIR-Dental School, University of Turin, 10126 Turin, Italy; matteo.visca@edu.unito.it (M.V.); federico.mussano@unito.it (F.M.)

**Keywords:** senescence, MSCs, rejuvenation

## Abstract

Mesenchymal stem/stromal cells (MSCs) are involved in the maintenance and regeneration of a large variety of tissues due to their stemness and multi-lineage differentiation capability. Harnessing these advantageous features, a flurry of clinical trials have focused on MSCs to treat different pathologies, but only few protocols have received regulatory approval so far. Among the various causes hindering MSCs’ efficacy is the emergence of cellular senescence, which has been correlated with specific characteristics, such as morphological and epigenetic alterations, DNA damage, ROS production, mitochondrial dysfunction, telomere shortening, non-coding RNAs, loss of proteostasis, and a peculiar senescence-associated secretory phenotype. Several strategies have been investigated for delaying or even hopefully reverting the onset of senescence, as assessed by the senescent phenotype of MSCs. Here, the authors reviewed the most updated literature on the potential causes of senescence, with a particular emphasis on the current and future therapeutic approaches aimed at reverting senescence and/or extending the functional lifespan of stem cells.

## 1. Background

Mesenchymal stem cells, also known as mesenchymal stromal cells (MSCs), are multipotent non-hematopoietic stem cells that represent a reservoir indispensable for the homeostatic maintenance and regeneration of a large variety of tissues throughout their individual lifespans, owing to their capacity of keeping their “stemness” while differentiating into the required histotype [1]. Since they are ubiquitous in the human body [2], MSCs have been defined through “minimal criteria” by Dominici et al. in 2006 as follows: (a) tissue culture plastic-adherent; (b) positive (≥95%) for surface antigen markers CD105, CD90, and CD73 while also being negative (≤2%) for CD45, CD34, CD14 or CD11b, CD79α or CD19, and HLA-DR; and (c) capable of differentiating to adipocytes, chondroblasts, and osteoblasts [3]. MSCs are also well recognized for their unique immunomodulatory functions, which allow them to exert regulatory activity on the immune microenvironment. Specifically, MSCs have been demonstrated to inhibit the proliferation of T cells and immune defense responses, underscoring their immunosuppressive properties. 

In the human body, there are different sources of MSCs: bone marrow (BM), umbilical cord, and adipose tissue are particularly rich in MSCs, but the oral cavity also harbors a variety of MSCs [4]. In the past decade, about 77% of clinical trials dealing with MSCs regarded BMSCs and ASCs [5]. Notwithstanding the remarkable number of studies available, only a few MSC-based protocols have received regulatory approval [6]. Among the possible causes of this shortcoming are cells’ heterogeneity, considerably limiting the standardization of therapies and the onset of senescence [7]. Recent studies report that senescent MSCs show reduced regenerative potential, impaired migration, and altered paracrine secretion, promoting tissue damage [8]. Transplanted senescent cells exhibit less efficient therapeutic potential function and may be detrimental [9], posing a major issue for MSC treatments. Thus, preventing, or at least reducing, stem cell senescence is emerging as a paramount task to improve the efficacy of future clinical applications. 

## 2. Senescence Features of MSCs

Cellular senescence regards properly mitotic cells and is usually defined as a “cell state triggered by stressful insults and certain physiological processes, characterized by a prolonged and generally irreversible cell-cycle arrest with secretory features, macromolecular damage, and altered metabolism” [10]. Senescent cells are resistant to apoptosis and show alterations in cellular and tissue repair mechanisms [11]. For instance, autophagy, a highly conserved evolutionary process controlling cellular homeostasis, has been associated with aging and a range of pathological conditions [12,13] since it is essential for the degradation and recycling of senescent or damaged organelles and macromolecules via lysosomal pathways [14]. As a physiological homeostatic mechanism, senescence is implicated in tissue repair [12], while its impairment leads to inflammatory diseases associated with aging such as osteoarthritis and Alzheimer’s disease [15,16]. Senescence is important to control cell growth as it protects against tumorigenesis by preventing cells with stress-induced damage from entering the replicative cycle [17]. 

**Figure 1:**
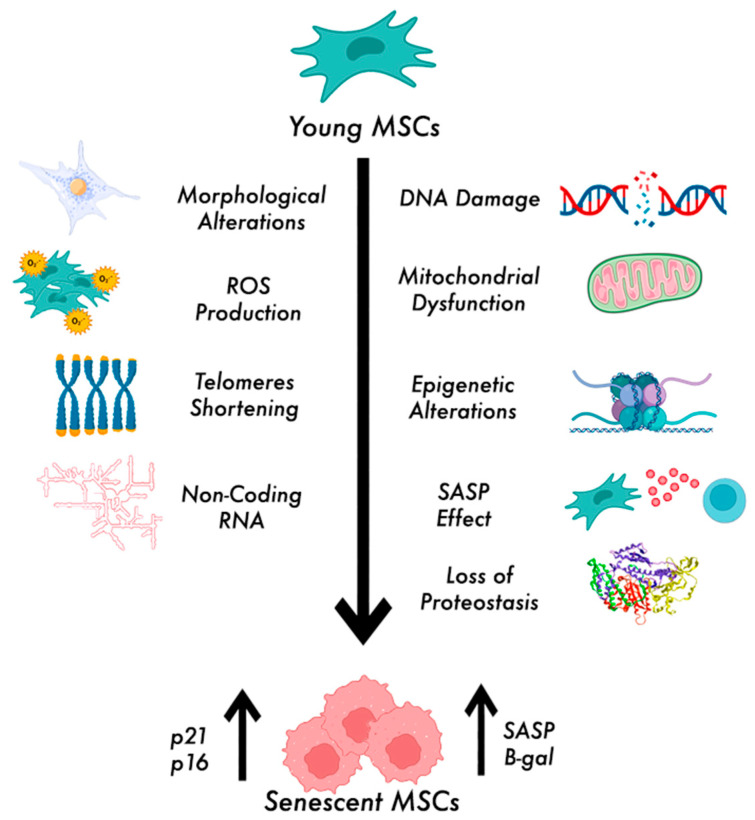
Features of senescent MSCs. Senescence is associated with different mechanisms, such as morphological alterations, ROS production, DNA damage, mitochondrial dysfunction, telomere shortening, epigenetic modifications, non-coding RNA control, loss of proteostasis, and SASP production, which lead to typical phenotype of senescent MSCs, expressing p16, p21, β-gal, and SASP.

In the following paragraphs, we will focus more carefully on different features of MSC senescence, which are shown in Figure 1.

### 2.1. Surface Markers and Morphological Changes

**Figure 2:**
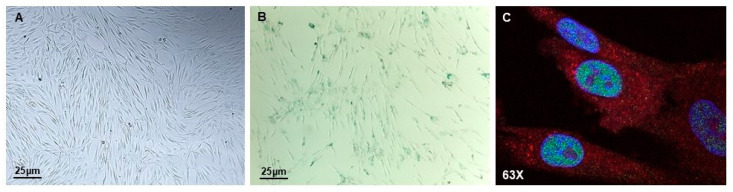
Senescent MSCs. The images show PDLSCs isolated from periodontitis patients, cultured in vitro, with a typical spindle-shaped cell body (**A**) and stained positive for β-gal (**B**). A confocal analysis showing nuclei (blue) and the co-expression of p21 (green) and OCT4 (red); magnification: 63× (**C**).

In cell culture, MSCs have a morphology characterized by a spindle-shaped cell body with a few long, thin processes, a large nucleus, and a differentiated nucleolus (Figure 2A). Senescent MSCs modify their morphology, becoming more enlarged and flatted with small nuclei, a granular cytoplasm, augmented actin stress fibers and losing their spindle-shaped characteristics. The senescence-associated morphological changes have been linked to the status of the scaffolding protein caveolin-1 (CAV-1), which is thought to regulate actin stress fiber formation [18]. It is widely recognized that MSCs tend to differentiate preferentially towards adipose tissue during senescence. Peroxisome proliferator-activated receptor-γ (PPAR-γ), an adipogenesis transcription factor, increases during senescence, thereby directing MSC differentiation towards adipogenesis. In contrast, Wnt/β-catenin (WNT) signaling can inhibit adipogenesis and promote MSCs’ differentiation into osteoblasts, positioning WNT signaling as a critical element in the adipogenesis and osteogenesis balance, as well as in regulating cellular senescence [19]. WNT signaling is downregulated according to age, but an increased activation of WNT signaling can also induce MSCs’ senescence. 

In 1961, Hayflick and Moorhead described cellular senescence for the first time by reporting that human diploid fibroblasts derived from fetuses lost the ability to proliferate and degenerate after about 50 sub-cultivations and one year in culture [20]. Prolonged culture can lead to cellular senescence, which can significantly impair MSCs’ function in terms of their immunomodulatory function, proliferation rate, and expression of surface antigens, even prior to their expected replicative limit. Indeed, senescent MSCs downregulate the expression of alkaline phosphatase (ALP) and osteocalcin, correlating with the observed decrease efficiency in bone formation during long-term culture [19]. STRO-1, CD106, and CD146 were expressed by MSCs and downregulated in vitro and/or in vivo during senescence, allowing for the identification of senescent surface markers on MSCs. Positive staining for SA-β-gal activity is universally known as a feature of senescent cells (Figure 2B); indeed, β-gal activity in senescent cells is significantly enhanced due to the increased lysosomal activity and altered cytosolic pH [21], along with the persistent expression of p53, p21, and p16 proteins (Figure 2C) [22].

CD264+ MSCs display elevated SA-β-gal activity, decreased proliferation rates, and reduced differentiation potential, whereas CD295+ MSCs exhibit lower proliferative capacity, with CD295/LEPR specifically identifying a distinct subpopulation of cells undergoing apoptosis [23]. The concomitant decrease in the adhesion molecule CD146 and the higher expression of decoy TRAIL receptor CD264 have been associated with senescent MSCs [24]. Recently, autofluorescence has also been reported as a feature of senescence [25]. Finally, senescent MSCs display nuclear changes, such as the loss of Lamin B1, that even precede SASP and SA-β-gal activity and the onset of senescence-associated heterochromatic foci (SAHF). These punctate nuclear structures are probably condensed chromosomes that depend on p53 and Rb activation [26].

### 2.2. ROS Production and DNA Damage

MSCs accumulate DNA damage over time because of various stressors, such as genotoxic agents, nutrient deprivation, hypoxia, and mitochondrial dysfunction [10]. Both endogenous (oncogene activation, chronic inflammation, and oxidative stress driven by the accumulation of reactive oxygen species (ROS) and exogenous mutagens (such as irradiation) are known contributors to DNA damage [27]. ROS, such as peroxide, superoxide, and hydroxyl radical, are normally found in cells and play a fundamental role in cellular signaling to perform physiological functions. Nonetheless, high ROS concentration drives MSCs toward senescence through different pathological mechanisms. Cell cycle arrest in senescent MSCs is influenced by oxidative stress, such as the release of DNA-damaging ROS [28]. Natural defense mechanisms against ROS, which are physiological products of the aerobic metabolism, may not be sufficient to avoid senescence owing to oxidative stress. Generally, the regulation of ROS levels and oxidative repair mechanisms become less efficient with aging, leading to ROS accumulation, molecular damage, and ultimately MSC senescence [29]. Senescent MSCs, in turn, produce more ROS, creating a positive feedback loop in which the more ROS are present, the worse the senescence and the consequent molecular damage [30]. Antioxidants scavenge local ROS, delaying the onset of senescence; when aged MSCs are treated with reduced glutathione and melatonin, they reacquire early-passage stemness and migratory capabilities, lowering the expression of p16 and p53 [31]. 

Consistently, hypoxic culture promotes the inhibition of MSCs’ senescence with the retention of stem cell properties compared to normoxia, and more importantly, without increasing tumorigenicity. ROS can stimulate the MAPK pathway both directly and indirectly, and the inhibition of the p38 MAPK pathway has been shown to prevent senescence [32]. Indeed, the activation of this pathway promotes p53 phosphorylation, allowing ROS to induce senescence. Additionally, the phosphatidylinositol-3-kinase (PI3K)-protein kinase B (AKT) signaling pathway is also implicated in MSC senescence [33,34]. This activation subsequently induces the transcription of target genes, such as the mechanistic target of rapamycin 1 (mTORC1), forkhead box protein O3 (FOXO3), and p53, ultimately leading to MSC senescence [35]. Since hypoxic culture conditions decrease the senescent phenotype by mimicking the biological niche of MSCs, hypoxic pre-conditioning was proposed to rejuvenate MSCs before transplantation [36]. 

The DNA damage response network regulates cell cycle arrest, which is a hallmark of MSC senescence and is primarily governed by the p21^CIP1/WAF1^ and p16 ^INK4A^ signaling pathways, which are crucial regulators of genes involved in the control of cell growth and proliferation, ultimately leading to cell cycle arrest and the prevention of re-entry into the cell cycle by damaged and/or senescent cells [37]. p21 is a cyclin-dependent kinase (CDK) inhibitor that is induced by DNA damage and transcriptionally activated by p53. The expression levels of p16 and p21 are upregulated both in vitro and in vivo during cellular senescence [38]. The activation of p21 reduces the phosphorylation of retinoblastoma protein (RB), allowing RB to retain its function and continue to suppress the E2 transcription factor [39,40]. Cell cycle withdrawal has also been described in quiescence, which is reversible upon proper stimulation, and in terminal differentiation with apparent differences. In particular circumstances, such as in tumors, a subset of malignant senescent cells upregulate stemness genes and can re-enter the cell cycle, inducing a much more aggressive phenotype of tumor cells [41,42]. 

An abundance of ROS not only triggers the DNA damage response, but also induces a range of other detrimental effects, such as mitochondrial dysfunction, telomere attrition, and protein degradation, which contribute to cellular senescence.

### 2.3. Mitochondrial Dysfunction

Mitochondria exert a crucial role in cellular senescence as they are essential for cellular respiration, and their dysfunction is associated with abnormal NAD+/NADH and ATP/ADP ratios, which are associated with MSCs’ senescence. An imbalance in the ATP/ADP ratio may activate 5ʹ-AMP-activated protein kinase (AMPK), inducing senescence through the p53/p21 pathway. Mitophagy regulates the selective degradation of damaged or dysfunctional mitochondria. Insufficient mitophagy has been implicated in senescence-related cellular injuries through the accumulation of damaged mitochondria and the consequent metabolic dysfunction. Recent findings indicate that reduced PARKIN (an E3 ubiquitin ligase involved in mitophagy) translocation to damaged mitochondria, through the p53 pathway, leads to defective mitophagy, hindering the clearance of damaged mitochondria. Additionally, ROS accumulation and sirtuin (SIRT) deficiency have been shown to inhibit PARKIN-mediated mitophagy, further contributing to cellular senescence [43,44].

It is established that elevated ROS levels cause increased mitochondrial DNA (mtDNA) damage, and due to the lack of efficient repair mechanisms, mtDNA is more susceptible to mutations than nuclear DNA. Age-related mtDNA abnormalities contribute to an augment of ROS production, creating a vicious cycle that disrupts the balance between ROS and antioxidants [45,46]. Another theory suggests that ROS-induced DNA damage stimulates mitochondrial biogenesis, leading to a higher number of mitochondria and further increasing mitochondrial ROS production, thus perpetuating another vicious cycle [47]. 

### 2.4. Telomeres Shortening

“Mammalian telomeres are composed of tandem repeats of TTAGGGn DNA sequences associated with a six-member protein Shelterin complex that facilitates the formation of a lariat-like structure (the t-loop) to shield the exposed chromosome ends of telomeric DNA from DNA damage machinery” [48]. Progressive telomere shortening occurs in all dividing normal somatic cells, and even stem cells, although not at the same rate [49]. This phenomenon originates from the incomplete lagging-strand DNA synthesis during DNA replication, owing to the inability of standard DNA polymerase to replicate fully linear DNA in the absence of telomerase [50], a ribonucleoprotein enzyme (whose catalytic component is represented by the telomerase reverse transcriptase TERT, named hTERT in humans) that is responsible for telomere elongation. 

When telomere shortening reaches a critical length, due to the decrease in telomere capping proteins, DNA damage response pathways arrest cell proliferation [51]. If telomere-binding proteins are sufficient to inhibit DNA repair and avoid fusions [52], a permanent DNA damage-induced proliferative arrest occurs, initiating cell senescence. Activation of the DNA damage response at telomeres leads to the formation of telomere-associated DDR foci (TAFs), which are markers of cellular senescence in cultured cells [50]. In their inspiring magistral review, Rosiello et al. [50] proposed a comprehensive perspective on how persistent DRR at the telomers may explain both replicative cellular senescence caused by critically short telomere and the senescence-like state occurring in non-replicating cells. Hence, the somehow simplistic concept of telomere shortening (classically describing replicative senescence) has been overcome by a more complex “telomere-centric” etiopathological mechanism unifying several aging hallmarks, which is also in agreement with the findings of Chakravarti et al. [53].

### 2.5. Epigenetic Alterations

Histone modification and DNA methylation are epigenetic events implicated in cellular senescence and may drive alterations associated with MSC senescence. Histone modification plays a significant role in influencing the transcriptional activities of nearby genomic regions. Histone deacetylases (HDACs), a group of enzymes that modulate histone acetylation levels, determine whether chromatin is in a relaxed euchromatin state associated with an open configuration and active transcription or a condensed heterochromatin state and an inactive conformation. HDAC inhibitors can accelerate aging by activating the transcription of p21 through the acetylation of histones H3 and H4 [54]. Moreover, HDAC inhibitors can regulate the cellular senescence gene CDKN2A via multiple microRNAs [54]. During senescence, EZH2, the histone lysine methyltransferase that allows the trimethylation of lysine 27 on histone H3 (H3K27me3), is downregulated, resulting in the loss of H3K27me3 at the *CDKN2A* locus with the consequent upregulation of p16 and the activation of genes of the senescence-associated secretory phenotype (SASP) [55]. Indeed, the role of EZH2 seems broader as its downregulation was proven to induce senescence by activating a DDR in proliferating cells before the reduction in the levels of H3K27me3 marks [55]. DNA methylation rules the gene expression inactivation of the X-chromosome, transposon silencing, cell specification, and cellular identity maintenance [56,57].

Changes in the DNA methylation profile also regulate the self-renewal and differentiation of adult stem cells [58]. During differentiation, DNA methylation increases, leading to the silencing of pluripotency-associated genes and other genes involved in non-specific cell type differentiation [59]. The methylation status is generally regulated by DNA methyltransferases (DNMTs), which include three major members: DNMT1, which maintains the methylation pattern during DNA replication, and DNMT3A and DNMT3B, which are responsible for de novo methylation. The expression levels of DNMT1 and DNMT3B are significantly reduced during senescence [60].

### 2.6. Senescence-Associated Secretory Phenotype (SASP)

Senescent MSCs can be distinguished from healthy cells based on their immunogenicity [61]. Senescent cells exhibit SASP, i.e., a multitude of secretory factors, including inflammatory and matrix-modeling signaling molecules, released with autocrine and paracrine effects. The SASP mediates the conversion of the anti-inflammatory MSC phenotype to the proinflammatory one [62]. Senescent MSCs increase the production of many cytokines and factors, including IL-1, IL-3, IL-4, IL-6, IL-8, IL-17, interferon-β (IFN- β), epidermal growth factor (EGF), fibroblast growth factor-2 (FGF-2), FGF-4, and FGF-8, hepatocyte growth factor (HGF), insulin growth factor-1 (IGF-1), platelet-derived growth factor (PDGF), transforming growth factor-β (TGF-β), and vascular endothelial growth factors (VEGFs) [24]. Among the factors contributing to the SASP of aged MSCs are the persistent activation of TLR signaling [63] and the increased adipogenesis associated with aging [64].

SASP and the immune system have a complex interaction. While SASP factors are known to recruit immune cells, such as macrophages, T lymphocytes, natural killer cells, and neutrophils, which promote the clearance of senescent cells [65], aged MSCs exhibit an altered immune-suppressive function. In physiological conditions, MSCs promote the polarization of macrophages towards an anti-inflammatory phenotype, M2 [66]; conversely, aged MSCs promote a shift in macrophage from the M2 to M1 (pro-inflammatory) phenotype, inducing inflammation [67]. MSCs exert strong immunosuppressive action on T cells, while aged MSCs show an impaired capability to reduce the proliferation of T cells [68,69].

SASP factors can act on neighboring cells through paracrine mechanisms to accelerate senescence. Indeed, non-coding RNA can be both contained in SASP and secreted in exosomes, contributing to the induction of cellular senescence in young cells [70,71]. Exosomes play a dual critical role in aging and cellular senescence, either promoting aging through SASP or exerting anti-aging effects when secreted by young and healthy MSCs [72]. Indeed, extracellular vesicles (EVs) from young MSCs have also been shown to rejuvenate hematopoietic stem cells by transferring autophagy-related and lineage commitment-related mRNAs [73]. EVs can transport pericentromeric non-coding RNAs into neighboring cells, impairing the DNA binding of the CCCTC-binding factor to modify chromosomal accessibility, stimulating an SASP-like inflammatory response [74]. 

Recent studies have highlighted the significant role of non-coding RNA in the aging and differentiation of MSCs [75]. For instance, miR-486-5p and miRNA-155-5p are elevated in the serum and MSCs of aged human donors but not in those of younger donors [70,76]. miR-486-5p inhibits osteogenic and adipogenic differentiation and induces MSC senescence through the targeted inhibition of SIRT1 expression [76]. miRNA-155-5p induces MSC senescence through mitochondrial dysfunction in an AMPK-dependent manner. Its inhibition has been shown to mitigate cardiac impairment in an aged mouse model, suggesting a potential target for rejuvenating MSCs [77]. The expression of miR-335 in MSCs derived from older subjects has been shown to affect MSC senescence by inhibiting AP-1 activity [78]. MiR-1292 has been shown to regulate senescence and osteogenesis through the Wnt/β-catenin signaling pathway [79].

The miR-17-92 cluster is mainly an oncogenic miRNA cluster [80] that is decreased in senescent adipose tissue-derived MSCs, contributing to the increase in p21 expression, which is associated with senescence. Moreover, the miR-17-92 cluster also affected the oxidative homeostasis of MSCs by regulating thioredoxin-interacting protein, which is induced by oxidative stress and inhibits the antioxidant protein thioredoxin [81].

### 2.7. Loss of Proteostasis

The proteostasis network is a macromolecular system that coordinates the synthesis, folding, disaggregation, and degradation of proteins. The proteasome system is also responsible for removing normal and damaged proteins, participating in the aging mechanism and longevity regulation. Thus, the loss of proteostasis has deep consequences for aging and age-related diseases. During aging, the aggregation of proteostasis network components can elicit reduced folding capacity, aberrant transcriptional procedures, and the accumulation of misfolded species [82]. Proteostasis impairment also depends on the aging-related pause of ribosome-associated quality control [83]. The autophagy–lysosomal system and the ubiquitin–proteasomal system (UPS) are two crucial pathways for removing misfolded proteins and damaged organelles in the muscles, as revied by Fernando et al. [84]. Aging often shifts the balance between the protein lifecycle in organisms, leading to pathologic conditions. For instance, UPS dysregulation is associated with the aging process and several aging-related diseases in mammals [85]. 

## 3. MSCs’ Rejuvenation Strategies

Existing research has focused on new therapeutic strategies based on molecules capable of preventing the senescent status of MSCs. MSCs derived from elder donors exhibit reduced proliferation, differentiation capacities, and stemness compared to those from younger donors, indicating an inverse relationship between the functional capacity of MSCs and their divisional paths. Moreover, it is relevant and beneficial to delay senescence as much as possible, since a remarkable cell expansion is mandatory in regenerative protocols to treat a person who needs one to two million cells per kilogram of body mass, and the yield of MSCs in their niche is estimated to be as low as 1 in every 50 million cells [86]. Therefore, understanding the molecular mechanisms underlying MSC senescence is crucial for optimizing the therapeutic potential of MSCs.

A series of approaches are described in the following section focusing on the techniques that are likely to achieve clinical translation (Table 1).

### 3.1. Anti-Aging Molecules/Drugs

#### 3.1.1. ROS Reduction Through Antioxidants

Ascorbic acid is a prototypical antioxidant and an mTOR signaling inhibitor. It has been proven to be capable of greatly extending the MSC expansion limit, inhibiting ROS production through AKT/mTOR [87].

The N-acetyl derivative of the amino acid l-cysteine, N-acetyl-L-cysteine (NAC), has a well-established reducing activity, behaving like a reduced glutathione (GSH) precursor (by breaking thiolated proteins and releasing free thiols), which is a substrate of many antioxidant enzymes, and it can also directly act as an antioxidant. Notably, in the case of the significant depletion of endogenous GSH, NAC can act as a direct antioxidant for some oxidant species such as NO_2_ and hydrogen oxide radicals [88]. Indeed, it has been shown that MSCs, depleted of GSH through a specific inhibitor, died for apoptosis due to H_2_O_2_-induced oxidative stress [89]. During the procedure of a bone marrow MSC transplant in a mouse model, the generation of oxidative stress, which causes inflammation, represents a critical issue for the effectiveness of cell therapies. Thus, the use of NAC to inhibit this inflammatory process was investigated, showing that the pre-treatment of MSCs with NAC induced a significant increase in intracellular GSH levels, preventing oxidative stress, reducing apoptosis and senescence, and promoting osteodifferentiation [90].

Supplementation with vitamin E, such as the synthetic compound α-tocopherol-acetate (α-TOA), has been shown to help maintain the proliferative capability of MSCs. It seems that α-TOA exhibits a low-oxygen-concentration-mimicking effect, reducing mitochondrial oxygen consumption. In an in vivo animal model, supplementation with vitamin E guarantees levels of α-TOA which can sustain MSCs [91].

MSC senescence can also be addressed by reducing ROS production with lactoferrin. When senescent human MSCs, induced by hydrogen peroxide exposure, were treated with lactoferrin, the protein effectively suppressed hydrogen peroxide-induced intercellular ROS and apoptosis [92]. This suggests that lactoferrin holds promise as an antioxidant and a potential enhancer of the immunomodulatory potency of MSCs, mitigating the senescence effects triggered by ROS.

Moreover, various strategies have been developed to mitigate ROS-mediated oxidative stress in MSCs, employing drugs such as metformin [93], 5-azacytidine [94], and resveratrol [95]. Metformin is being trialed in humans as the first geroprotective drug, and in adipose-derived MSCs, it reduced replicative senescence likely owing to its ROS scavenging activity [96], which is consistent with the reported activation of AMP-activated protein kinase implicated in mitochondrial homeostasis. Indeed, cell rejuvenation is possibly attained by restoring mitochondrial function. High levels of scientific evidence regarding their antioxidant properties are available, although not specifically in MSCs, for fullerol [97], fucoidan [98], and carvediol [99]. The possible translation of the advantageous properties of pigment epithelium-derived factor (PEDF) (a serpin member of the superfamily of serine protease inhibitors [100], that is prevalently studied in the ophthalmic field) to MSCs is also encouraged. Anecdotally, exendin-4 preconditioning was reported to attenuate apoptosis induced by H_2_O_2_ in ASCs [101], similarly to Cirsium setidens [102]. Furthermore, exposure to basic fibroblast growth factor (bFGF) was described as beneficial in reducing liver ischemia–reperfusion injury owing to its antioxidant properties [103]. Ginsenoside RG1 has been shown to extend the lifespan, enhance the proliferation, and promote the colony formation of bone marrow stromal cells. The treatment of bone marrow mononuclear cells with ginsenoside Rg1 reduced apoptotic- and SA-β-gal-positive cells, accompanied with decreased ROS generation and improved colony-forming capacity [104].

Although all these agents have demonstrated effectiveness in reducing senescence in MSCs, caution is suggested as their prolonged usage may affect differentiation capacity. Indeed, at low intrinsic levels, ROS are beneficial for MSCs’ osteogenesis, and their excessive removal can result in a paradoxical induction of senescence in proliferating cells [105] as well as in the suppression of adipogenic potential [106].

Finally, among nutraceuticals (which are naturally occurring biomolecules found in food and other natural sources) that have demonstrated anti-senescence effects [107,108], polyphenols exhibit significant antioxidant and anti-inflammatory properties. Polyphenols are broadly categorized into flavonoids and non-flavonoids and have been shown to exert anti-SASP effects by downregulating oxidative stress and inflammatory pathways. It has been reported that MSCs’ stemness could be promoted by some food-derived nutrients [108,109].

#### 3.1.2. Autophagy Regulation

The autophagy–lysosomal pathway is important in maintaining cellular equilibrium, and targeting this pathway has emerged as a promising anti-aging strategy by either suppressing cellular senescence or inducing apoptosis in senescent cells. Regulating the autophagy level is also a way to rejuvenate senescent MSCs. Treatment with the autophagy inhibitor rapamycin considerably downregulated SASP in senescent MSCs. Among the most promising anti-senescent drugs, rapamycin is a potent inhibitor of the mechanistic Target Of Rapamycin Complex One (mTORC1) protein kinase, which showed anti-aging properties such as the potential to restore differentiation and proliferation, rescue nuclear membrane deformation, revert morphological changes, and activate SA-β-gal and p53/p21 expression in diverse model systems, including stem cells derived from a progeroid mouse model [110]. It is noteworthy that these beneficial effects rely on the inhibition of mTORC1, while most unwanted side effects are due to the inhibition of mTORC2, which has paved the way for exploring a therapeutic strategy selectively targeting mTORC1 [111]. Moreover, the overexpression of macrophage migration inhibitory factor (MIF) has been demonstrated to rejuvenate aged MSCs by activating autophagy [112]. Different clinical trials test the safety and efficacy of rapamycin in attenuating the aging process, but to date, no human data have supported its use as a geroprotector, which is a remarkable translational knowledge gap [111]. 

#### 3.1.3. Mitochondria Targeting

As regards the drugs targeting mitochondria with senotherapeutic potential, besides the antioxidant mitoquinone [113], it is worth mentioning melatonin. When compared to untreated controls, melatonin-treated senescent MSCs seem to possess enhanced therapeutic potential, sustaining better ischemic recovery and neo-vascularization in murine models [114]. From a molecular point of view, melatonin increases sirtuin 1 (SIRT1) expression while inhibiting ROS accumulation, and it also activates mitophagy which can clear damaged mitochondria [114]. Sirtuins, a family of NAD-dependent HDACs, regulate glucose and insulin metabolism, protein homeostasis, and circadian rhythms, resulting in the pivotal delay of cellular senescence. To date, three sirtuin members (SIRT1, SIRT3, and SIRT6) have been identified as potential anti-aging factors. 

Likewise, other molecules promoting SIRT1 activity, such as resveratrol or nicotinamide adenine dinucleotide (NAD), ameliorate the “stemness” properties of early-passage MSCs while rescuing the functional impairment of senescent cells [115]. Unfortunately, this outcome is only achieved at high drug concentrations or through prolonged use. To counteract this pitfall, Wang et al. recently proposed a “targeting nanoplatform with a strong affinity for senescent MSCs through conjugation with anti-Kremen 1”, which may be useful in future nano-therapeutics [116].

The precise mechanisms underlying the anti-aging effects of SIRT1 and SIRT3 are not fully elucidated but are believed to involve the maintenance of genomic stability. The depletion of SIRT3 accelerates aging and inhibits MSC differentiation into osteoblasts and adipocytes. In late-passage MSCs, the overexpression of SIRT3 can reduce oxidative stress, restore their differentiation capacity, and mitigate senescence [117]. SIRT6 specifically deacetylates histone lysine residues H3K9, H3K18, and H3K56, which maintain genomic integrity through the formation of a repressive heterochromatin structure [118,119]. 

#### 3.1.4. DNA demethylation

Epigenetics refers to heritable changes in gene expression that occur during cell division without alterations in the DNA sequence itself [120]. Chemical modifications to the DNA molecule and associated histone proteins can influence the chromatin structure, thereby altering the accessibility of transcription factors to gene regulatory regions. These modifications are critical for maintaining cells in an undifferentiated state or guiding them toward specific cell fate decisions. DNA methylation is mediated by DNMT enzymes, with DNMT1 functioning as a maintenance methyltransferase, ensuring the propagation of methylation patterns at replication forks [121]. DNMT3A and DNMT3B are responsible for de novo DNA methylation, introducing methyl groups to previously unmethylated CpG sites [112]. Conversely, active DNA demethylation involves the oxidation of methylcytosine to hydroxymethylcytosine, a process catalyzed by Ten-eleven Translocation (TET) enzymes [122]. DNMT inhibitors are a category of anti-senescence drugs that have been acquiring interest in the past few years. RG108 is a small molecule specifically designed to inhibit the catalytic activity of DNMTs [123]. Its demonstrated capacity to reactivate several tumor suppressor genes, coupled with its lack of cytotoxicity [123,124], positions RG108 as a promising candidate for epigenetic modulation therapies in regenerative protocols. The effect of RG108 has been studied in the treatment of aging-related diseases, demonstrating its beneficial effects in restoring aberrant DNA methylation patterns in human MSCs [125]. Subsequently, it was further shown that the senescence phenotype driven by excessive DNMT expression observed in amyotrophic lateral sclerosis (ALS) could be mitigated by RG108 treatment [126]. The authors proposed that RG108 may enhance the therapeutic efficacy of hMSCs in stem cell therapy, as it was shown to restore cell function by improving stem cell potency, enhancing cell migration, providing protection against oxidative stress, and reducing senescence. Also, on bone marrow derived-MSCs of swine origin, RG108 exerted an effect on pluripotency gene expression, apoptosis, and senescence [127]. These data were confirmed in Periodontal Ligament-Derived Stem Cells (PDLSCs) derived from patients with periodontitis, which show some senescent characteristics. Indeed, treatment with RG108 was able to suppress the senescent phenotype of PDLSCs harvested from patients with periodontitis by inducing the downregulation of p16 and p21, increasing the expression of stemness genes, such as OCT4, and rescuing multi-differentiation ability [128].

#### 3.1.5. Senolytics: The Elimination of Senescent Cells

Senescent MSCs are more resistant to apoptotic stimuli; thus, senolytic drugs effectively eliminate senescent cells by targeting their anti-apoptotic pathways, and they represent a further pharmacological strategy to treat many age-related diseases [129,130].

Proteomic and transcriptomic analyses identified many senescent cell anti-apoptotic pathways (SCAPs), which are more expressed in senescent than non-senescent cells and account for the apoptosis resistance of senescent cells [131].

The *BCL-2* gene family contains the main regulators of programmed cell death [132]; indeed, its members are upregulated during cellular senescence and downregulated or inhibited when promoting the apoptosis of senescent cells. Several BCL-2 inhibitors have been identified as promising senolytic agents [133,134,135], but their use is restricted to clinical trials since they can induce significant side effects [136]. Dasatinib, an FDA-approved anti-cancer drug, acts as a tyrosine kinase inhibitor, effectively reducing cell proliferation and migration while inducing apoptosis [137], and it kills senescent human fat cell progenitors, while quercetin is more effective against senescent human endothelial cells and mouse bone marrow MSCs [131]. Senolytic flavonoids, such as quercetin and fisetin, induce apoptosis by blocking members of the BCL-2 family such BCLxL, as well as HIF-1 and other SCAP network elements [138]. Quercetin showed a wide range of biological activities, including interactions with specific isoforms of PI3K and members of the BCL-2 family. Moreover, quercetin alone or associated with Dasatinib has been proven to reduce the number of senescent mouse bone marrow MSCs [131]. Fisetin attenuates senescence markers while maintaining the differentiation potential of adipose-derived mesenchymal stem cells [139].

Other mechanisms of apoptotic resistance, dependent on different proteins or pathways, are activated in senescent cells; thus, new molecular targets have been developed for the development of therapies to clear senescent cells. Recently, p53 and its associated regulatory networks have emerged as other targets for the development of senolytic therapies. The transcription factor p53 has a fundamental role in various biological processes, such as the regulation of cell growth and apoptosis with both the initiation and maintenance of senescence, DNA repair, and cellular stress response [140].

The E3 ligase murine double minute 2 (MDM2) is a negative regulator of p53, promoting its degradation via the proteasome. As a small-molecule inhibitor of the MDM2/p53 interaction, RG7112 is used to restore p53 activity, inducing senolysis in senescent intervertebral disc cells, reducing SASP factors such as IFN-γ, IL-6, and CCL24 in vitro [141].

Ubiquitin-specific peptidase 7 (USP7) deubiquitinates MDM2, preventing its degradation via the ubiquitin–proteasome system, thus offering an alternative approach to stabilization and upregulation [142]. USP7 inhibitors have shown the ability to selectively eliminate senescent cells by reducing MDM2 levels and increasing p53 activity, ultimately leading to the induction of pro-apoptotic proteins [143].

Finally, proteolysis-targeting chimeras (PROTACs) are bifunctional molecules consisting of a ligand that binds a target protein and another ligand that recruits an E3 ubiquitin ligase connected by an optimized linker. PROTACs function by bringing the target protein and E3 ligase into close proximity, leading to the ubiquitination and subsequent degradation of the target protein by the ubiquitin–proteasome system [142]. Unlike traditional inhibitors which inhibit the activity of the target proteins, PROTACs act in a catalytic manner to degrade the target [144]. This approach has been applied to enhance the efficacy and specificity of senolytic therapies. For instance, a novel PROTAC-based senolytic, PZ15227, that binds to BCL-XL, demonstrated great efficacy in clearing senescent cells [145]. A second generation of senolytic drugs has been identified through high-throughput library screens and includes antibody−drug conjugates, lysosomal- and SA-β-gal-activated pro-drugs, sodium–potassium pump (Na+/K+-ATPase), and immune-mediated clearance by CAR T cells [135].

#### 3.1.6. Senomorphics: Suppression of MSC SASP

The initial hypothesis that MSCs contribute to tissue regeneration primarily through homing to injury sites and subsequent differentiation has been reconsidered due to the short implantation time of MSCs, which is typically insufficient to exert a substantial impact. It is now recognized that the survival rate of MSCs is less than 1% one week after systemic administration [146,147,148]. However, MSCs exert significant biological effects by promoting cell-to-cell interactions and cellular proliferation, largely through the secretion of paracrine factors such as growth factors and cytokines. Consequently, the MSC secretome may represent a novel avenue in medical biotechnology. Agents suppressing SASP production are called senomorphics, and they have been studied and are currently under investigation for their potential capability to prevent or treat age-related diseases and to extend healthspan [149]. Nonetheless, SASP inhibitors need continuous treatment to exert SASP suppression; thus, they could show more side effects compared to senolytic agents, which are taken on an intermittent schedule.

SASP inhibitors target pathways such as p38 MAPK, JAK/STAT, and interleukins. p38MAPK is an MAPK family member and regulates SASP; thus, p38MAPK inhibitors potently suppress SASP expression in senescent cells [150].

The JAK/STAT pathway regulates cytokine production [151]. JAK inhibitors repress the SASP of adipocyte progenitors by reducing systemic inflammation associated with aging [151].

Senescent normal and dysplastic oral keratinocytes release different levels of pro-inflammatory cytokines, and treatment with Rho kinase inhibitors, such as Y-27632, can reduce the dysfunctional levels of these cytokines without affecting permanent cell growth arrest [152].

**Table 1:** Therapeutic strategies for anti-MSC senescence. This table presents an overview of various therapeutic strategies aimed at retarding the onset of MSC senescence, which ranges from in vivo studies to clinical trials (Phase I–III). Each category outlines the targeted senescence feature and the mechanism of action and provides the current evidence level.

Therapeutic Strategy		Mechanism of Action	Level of Evidence
**ROS Reduction**			
	Ascorbic Acid	Inhibition of AKT/mTOR pathway	In Vivo [88]
	Lactoferrin	Suppression of hydrogen peroxide-induced intercellular ROS and apoptosis	In Vivo [93]
	Ginsenoside RG1	Reduction in SA-β-gal-positive cells and apoptotic cells, decreased ROS generation	In Vivo [105]
**Autophagy Regulation**			
	Rapamycin	Inhibition of mTORC1 protein kinase	Clinical Trial (I-II) [111]
	Metformin	ROS scavenging action, linked to activation of AMP-activated protein kinase	Clinical Trial (II-III) [153]
**Mitochondrial Targeting**			
	Melatonin	Increase in SIRT1 expression, inhibition of ROS accumulation, activation of mitophagy	Clinical Trial (I-II) [154,155]
**Nutraceutical**			
	Polyphenols	Antioxidant and anti-inflammatory properties (SASP reduction)	Clinical Trial (II-III) [155]
	Methylsulfonylmethane	Induction of MSC differentiation	Clinical Trial (I-II) [156]
**DNA demethylation**			
	RG108	DNMT inhibitors	In Vitro [157]
**Senolytic Drugs**			
	Dasatinib	BCL-2 family inhibitors	Clinical Trial (I-II) [158]
	Quercetin	BCL-2 family inhibitors	Clinical Trial (I-II) [158]
	Fisetin	BCL-2 family inhibitors	Clinical Trial (I) [159,160]
	RG7112	Restores p53 activity and reduces SASP factors	Clinical Trial (I) [161]
	USP7 inhibitors	USP7 inhibition, whichh leads to reduced MDM2 levels and increased p53 activity	In Vivo [143]
	PROTACs	Leads to E3 ubiquitination and subsequent degradation (PZ15227)	In Vivo [144]
**Senomorphic Drugs**			
	SASP Inhibitors	Suppression of p38 MAPK, JAK/STAT, and Rho/Kinase inhibitor pathways	Clinical Trial (I-II) [160]
**Non-coding RNAs**			
	MiR-335	Inhibition of AP-1 activity	In Vivo [78]
	MiR-1292	Regulation of Wnt/β-catenin signaling pathway	In Vivo [79]
	MiR-17-92	Contributes to increased p21 expression andaffects oxidative homeostasis	In Vivo [82]
	miR-486-5p	Induces MSC senescence by targeted inhibition of SIRT1 expression, induces mitochondrial dysfunction in AMPK-dependent manner	In Vivo [162]
	miRNA-155-5p	Induces mitochondrial dysfunction in AMPK-dependent manner	In Vivo [163]

## 4. Conclusions

Although MSCs are widely regarded as a powerful and safe therapeutic option, their actual translation to clinics has been limited to date. Key hurdles to MSC usage are cellular senescence and a natural response to accumulated stress-induced damage. To address this shortcoming, researchers have been working on several strategies capable of delaying or even hopefully reverting the senescent phenotypes of MSCs. In the present review, the authors focused on the recent advancements in the study of therapeutic anti-senescence approaches for MSCs. More studies are needed to elucidate the physiological mechanisms underpinning stem cell aging, aiming at developing novel methods that may extend the functional lifespan of stem cells in a safe, reproducible, effective, and tunable way. Indeed, the off-target effects of anti-senescent therapies may even include oncogenesis and thus require the uttermost attention to safety issues besides efficacy evidence.
